# Common Peroneal Nerve Schwannoma Presenting as a Popliteal Fossa Mass: A Case Report

**DOI:** 10.7759/cureus.111000

**Published:** 2026-06-16

**Authors:** Stavros Lykos, Dimitrios Pallis, Alexandros Chatzikyriakos, Nikolaos Paraskevopoulos, Ioannis Panagiotopoulos, Stamatios A Papadakis

**Affiliations:** 1 2nd Department of Orthopaedics, KAT General Hospital of Attica, Athens, GRC; 2 1st Department of Orthopaedics, Panagiotis & Aglaia Kyriakou Children's Hospital, Athens, GRC; 3 Department of Orthopaedic Surgery, National and Kapodistrian University of Athens School of Medicine, Athens, GRC; 4 B' Department of Orthopaedics, KAT General Hospital of Attica, Athens, GRC

**Keywords:** common peroneal nerve, lower limb neuropathy, peripheral nerve sheath tumor, popliteal fossa mass, schwannoma

## Abstract

Schwannomas are benign peripheral nerve sheath tumors originating from Schwann cells and represent the most common benign tumors of peripheral nerves. Involvement of the common peroneal nerve is uncommon and may clinically mimic cystic, vascular, or other soft-tissue lesions of the popliteal fossa, leading to delayed diagnosis. We report the case of a 32-year-old woman who presented with a painless swelling in the left popliteal fossa, followed by progressive paresthesia extending to the dorsum of the foot and great toe. Ultrasound demonstrated a well-defined hypoechoic lesion adjacent to the popliteal vein, while magnetic resonance imaging (MRI) revealed a well-circumscribed spindle-shaped mass arising from the common peroneal nerve, with imaging characteristics suggestive of a benign peripheral nerve sheath tumor. In April 2024, the patient underwent complete surgical excision under general anesthesia at the Second Department of Orthopedics, KAT General Hospital of Attica. Histopathological examination confirmed the diagnosis of schwannoma. The postoperative course was uneventful, with gradual resolution of sensory symptoms and complete recovery within three months. Although uncommon and frequently misdiagnosed, common peroneal nerve schwannomas should be considered in the differential diagnosis of persistent popliteal fossa masses or unexplained neuropathic symptoms of the lower limb. MRI plays a pivotal role in diagnosis and surgical planning, while careful, complete excision is associated with excellent neurological and functional outcomes.

## Introduction

Schwannomas, also referred to as neurilemmomas, are benign peripheral nerve sheath tumors originating from differentiated Schwann cells responsible for the myelination of peripheral nerve fibers [1]. They are typically encapsulated, slow-growing lesions that arise eccentrically from the parent nerve and often exhibit an indolent clinical course, which may result in delayed diagnosis [2]. Schwannomas account for approximately 5% of benign soft-tissue tumors and represent the most common benign tumors of peripheral nerves, most frequently occurring in the head and neck region and upper extremities, whereas lower limb involvement is considerably less common [3]. Schwannomas involving the common peroneal nerve are particularly rare and may pose a diagnostic challenge because of their deep anatomical location and nonspecific presentation, often mimicking more common conditions such as Baker’s cysts, lipomas, or vascular lesions of the popliteal fossa [4].

Histologically, schwannomas demonstrate a characteristic biphasic pattern consisting of Antoni A areas, composed of densely packed spindle cells with nuclear palisading (Verocay bodies), and Antoni B areas, which display a looser myxoid architecture [4]. Clinically, patients may present with a slowly enlarging palpable mass associated with pain, paresthesia, or dysesthesia along the distribution of the affected nerve, whereas motor deficits are less common and usually occur in advanced cases [2]. Magnetic resonance imaging (MRI) is the imaging modality of choice and typically demonstrates a well-circumscribed lesion with low-to-intermediate signal intensity on T1-weighted images and high signal intensity on T2-weighted images, facilitating diagnosis and surgical planning [4]. Definitive diagnosis is established histopathologically, while careful surgical excision remains the treatment of choice and is generally associated with favorable neurological and functional outcomes [2].

In this report, we present a rare case of schwannoma arising from the common peroneal nerve presenting as a popliteal fossa mass associated with progressive sensory symptoms, successfully treated with complete surgical excision and favorable neurological recovery.

## Case presentation

A 32-year-old woman initially presented to the emergency department of our hospital with swelling in the left popliteal fossa. The swelling had appeared without preceding trauma or signs of infection and was not associated with pain or limitation of knee movement. Clinical examination revealed mild edema in the popliteal region without erythema, local warmth, tenderness, or restriction of range of motion. Conservative management, including rest, cryotherapy, and outpatient reassessment after 10 days, was recommended; however, the patient did not return for follow-up.

Electrophysiological evaluation had been performed prior to referral and reportedly demonstrated findings consistent with common peroneal nerve dysfunction. However, the original study report was not available for review, and therefore, detailed results could not be included in this report.

Three months later, she re-presented to the outpatient clinic reporting numbness and tingling extending from the anterolateral aspect of the leg to the dorsum of the foot and great toe, findings consistent with sensory involvement of the common peroneal nerve. Motor function remained intact, with no evidence of weakness or gait disturbance.

Ultrasound examination of the soft tissues demonstrated a well-defined hypoechoic spindle-shaped lesion adjacent to the popliteal vein, which retained normal compressibility and blood flow (Figure 1).

**Figure 1:**
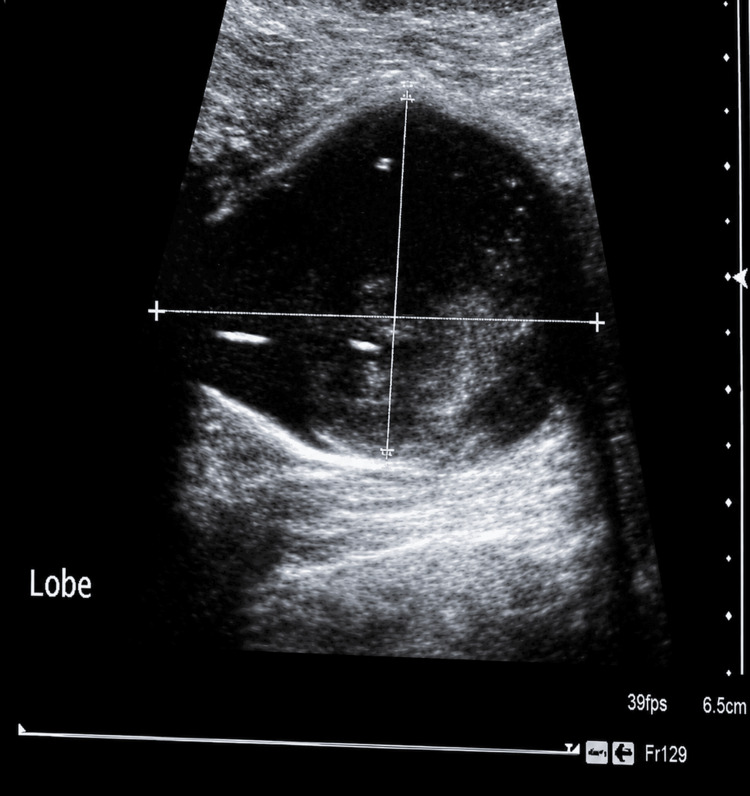
Ultrasound image showing a hypoechoic spindle-shaped lesion in contact with the popliteal vein, which retains normal flow and compressibility

MRI of the left knee revealed a well-circumscribed spindle-shaped lesion measuring 4.5 cm in maximum diameter, located within the adipose tissue of the popliteal fossa, between the semitendinosus, semimembranosus, and biceps femoris muscles at the level of the distal femoral diaphysis. The lesion followed the anatomical course of the common peroneal nerve and appeared to arise from it. It appeared isointense on T1-weighted sequences and hyperintense on T2-weighted sequences, with homogeneous contrast enhancement, findings suggestive of a benign peripheral nerve sheath tumor (Figures 2, 3) [5].

**Figure 2:**
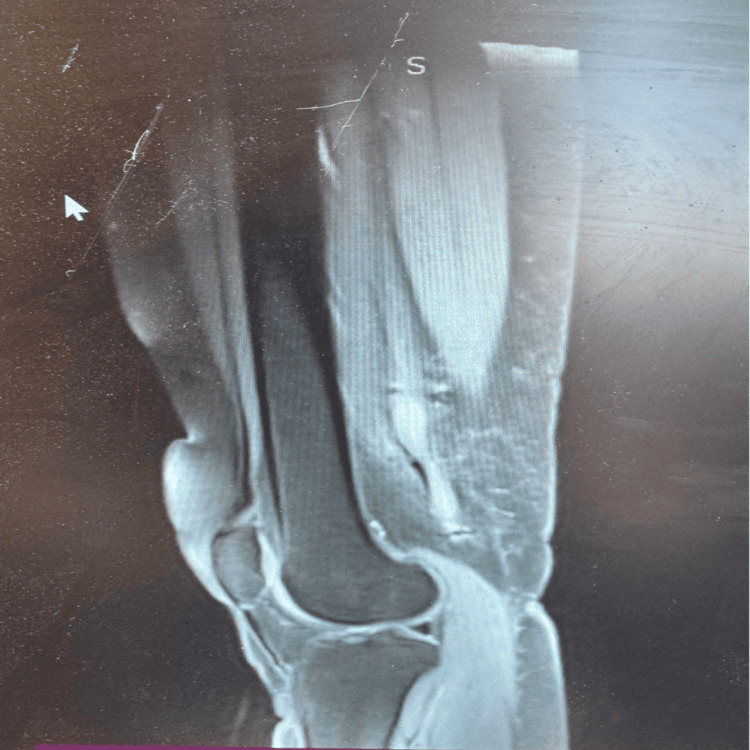
MRI view showing a spindle-shaped mass located within the adipocellular space between the semitendinosus, semimembranosus, and biceps femoris muscles, at the distal femoral diaphysis

**Figure 3:**
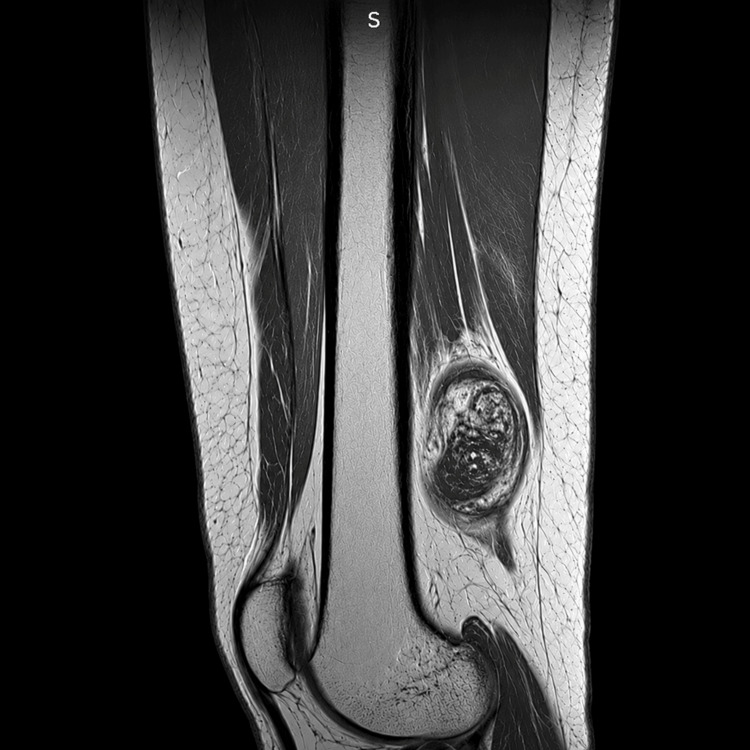
MRI view demonstrating well-defined margins and homogeneous enhancement of the lesion

In April 2024, the patient underwent surgical excision under general anesthesia and tourniquet control through a posterolateral approach. Intraoperatively, the lesion was found to be in close proximity to the common peroneal nerve, requiring meticulous and careful dissection to preserve neural continuity and prevent iatrogenic injury. Careful neurolysis and stepwise dissection were performed to adequately separate the mass from the nerve without compromising its integrity or function. Complete excision of the encapsulated mass was achieved without intraoperative complications.

Histopathological examination demonstrated a well-encapsulated tumor composed of spindle-shaped cells arranged in Antoni A and Antoni B areas, with nuclear palisading and Verocay bodies, confirming the diagnosis of schwannoma [6,7]. No mitotic activity, necrosis, or malignant features were identified.

The postoperative course was uneventful. The preoperative paresthesia gradually subsided, and complete sensory recovery was achieved within three months. At the two-year follow-up, the patient remained asymptomatic, with full knee range of motion and no evidence of recurrence.

Human ethics and consent

Written informed consent was obtained from the patient for publication of this case report and the accompanying images. According to institutional policy, ethics committee approval was not required for the publication of a single anonymized case report.

## Discussion

Schwannomas are the most common benign peripheral nerve sheath tumors, typically presenting as solitary, encapsulated lesions arising from Schwann cells [1,2]. They are characteristically slow-growing and eccentrically located relative to the parent nerve, which often results in a prolonged asymptomatic course and delayed clinical presentation [1,2].

These tumors account for approximately 5% of benign soft-tissue tumors and most commonly occur in the head and neck region and upper extremities, whereas lower limb involvement is relatively uncommon [3]. Within this distribution, schwannomas involving the common peroneal nerve are particularly rare and represent only a small fraction of reported cases. Their deep anatomical location and nonspecific clinical presentation contribute to diagnostic difficulty and delayed recognition [3,6-10].

Clinically, schwannomas typically present as slow-growing, well-defined masses that may be associated with pain, paresthesia, or dysesthesia along the distribution of the affected nerve [2,8]. A positive Tinel’s sign may be present and can assist in clinical suspicion of nerve involvement [1,8]. Motor deficits are uncommon and usually occur in long-standing cases due to chronic nerve compression [3,8]. In the present case, the initial presentation was limited to a painless popliteal swelling, with neurological symptoms developing later, consistent with previously reported patterns [7].

Because of their deep location in the popliteal fossa, common peroneal nerve schwannomas may mimic more frequent soft-tissue lesions such as Baker’s cysts, lipomas, or vascular abnormalities [4,5]. This often leads to misdiagnosis or delayed recognition, particularly when neurological symptoms are initially absent [4,5].

MRI is the imaging modality of choice for peripheral nerve sheath tumors and typically demonstrates a well-circumscribed lesion with low-to-intermediate signal intensity on T1-weighted images and high signal intensity on T2-weighted sequences, often with homogeneous enhancement [4]. Characteristic signs such as the “target sign” and “split-fat sign” may further support the diagnosis [4]. In the present case, MRI findings were highly suggestive of a benign peripheral nerve sheath tumor and were essential for surgical planning.

Compared with recently published reports of common peroneal nerve schwannomas, the present case highlights the diagnostic challenge posed by an initially painless popliteal fossa mass without neurological deficits, followed by delayed development of sensory symptoms. This clinical course underscores the importance of maintaining a high index of suspicion for peripheral nerve sheath tumors when evaluating persistent popliteal fossa lesions. Electrophysiological studies may provide complementary information regarding nerve dysfunction and can be useful in the preoperative evaluation of symptomatic patients. In selected cases, intraoperative neuromonitoring may also assist in preserving neural function during tumor excision, particularly when dissection from the parent nerve is technically demanding.

Definitive diagnosis is established histopathologically, demonstrating the characteristic Antoni A and Antoni B areas with nuclear palisading and Verocay bodies [4]. However, a strong preoperative diagnosis can often be made based on clinical and imaging findings.

Surgical excision remains the treatment of choice for symptomatic schwannomas and is associated with excellent functional outcomes [2,8]. Due to their encapsulated and eccentric growth pattern, these tumors can usually be excised while preserving nerve continuity and function [2]. Multiple studies have demonstrated high rates of complete symptom resolution and low incidence of permanent neurological deficits following surgery [6-10]. In particular, peroneal nerve schwannomas have been associated with favorable postoperative recovery, with most patients regaining full or near-full neurological function [6-10].

In the present case, complete surgical excision resulted in full resolution of sensory symptoms within three months, consistent with previously reported outcomes in the literature [3,7,8].

Limitations

This report has several limitations. First, it describes a single case, and therefore, the findings cannot be generalized. Second, although electrophysiological testing had reportedly demonstrated common peroneal nerve dysfunction, the original study report was unavailable for review, and detailed results could not be presented. Third, standardized neurological outcome measures and patient-reported outcome scores were not collected, limiting objective assessment of postoperative recovery. Finally, the imaging figures were obtained retrospectively from available clinical records, and image quality may not be optimal for detailed radiological assessment.

## Conclusions

Common peroneal nerve schwannomas are rare benign peripheral nerve sheath tumors that should be considered in the differential diagnosis of popliteal fossa masses and unexplained lower limb neuropathic symptoms. Due to their slow growth and nonspecific clinical presentation, they are frequently misdiagnosed, which may delay appropriate management. MRI is essential for accurate preoperative diagnosis and surgical planning. Careful surgical excision with preservation of neural structures is the treatment of choice and is associated with excellent functional and neurological outcomes, particularly when performed early.
